# *RPD3* and *UME6* are involved in the activation of *PDR5* transcription and pleiotropic drug resistance in ρ^0^ cells of *Saccharomyces cerevisiae*

**DOI:** 10.1186/s12866-021-02373-1

**Published:** 2021-11-09

**Authors:** Yoichi Yamada

**Affiliations:** Faculty of Biological Science and Technology, Institute of Science and Engineering, Kanazawa University, Kanazawa, 920-1164 Japan

**Keywords:** *Saccharomyces cerevisiae*, *UME6*, *RPD3*, Pleiotropic drug resistance, ρ^0^ cells, Retrograde signalling

## Abstract

**Background:**

In *Saccharomyces cerevisiae*, the retrograde signalling pathway is activated in ρ^0/−^ cells, which lack mitochondrial DNA. Within this pathway, the activation of the transcription factor Pdr3 induces transcription of the ATP-binding cassette (ABC) transporter gene, *PDR5,* and causes pleiotropic drug resistance (PDR). Although a histone deacetylase, Rpd3, is also required for cycloheximide resistance in ρ^0/−^ cells, it is currently unknown whether Rpd3 and its DNA binding partners, Ume6 and Ash1, are involved in the activation of *PDR5* transcription and PDR in ρ^0/−^ cells. This study investigated the roles of *RPD3*, *UME6*, and *ASH1* in the activation of *PDR5* transcription and PDR by retrograde signalling in ρ^0^ cells.

**Results:**

ρ^0^ cells in the *rpd3*∆ and *ume6*∆ strains, with the exception of the *ash1*∆ strain, were sensitive to fluconazole and cycloheximide. The *PDR5* mRNA levels in ρ^0^ cells of the *rpd3*∆ and *ume6*∆ strains were significantly reduced compared to the wild-type and *ash1*∆ strain. Transcriptional expression of *PDR5* was reduced in cycloheximide-exposed and unexposed ρ^0^ cells of the *ume6*∆ strain; the transcriptional positive response of *PDR5* to cycloheximide exposure was also impaired in this strain.

**Conclusions:**

*RPD3* and *UME6* are responsible for enhanced *PDR5* mRNA levels and PDR by retrograde signalling in ρ^0^ cells of *S. cerevisiae*.

**Supplementary Information:**

The online version contains supplementary material available at 10.1186/s12866-021-02373-1.

## Background

In the yeast, *Saccharomyces cerevisiae*, multidrug resistance can result from the overexpression of plasma membrane-localized ABC transporters, such as Pdr5, Snq2, and Yor1. Pdr5 is a major efflux pump of functionally and structurally unrelated antifungal drugs or compounds, such as fluconazole and cycloheximide [1]. Expression of *PDR5* can be induced by the paralogous Zn2Cys6 transcription factors, Pdr1 and Pdr3. Pdr1 and Pdr3 can form both homo- and heterodimers, and directly bind structurally diverse drugs and xenobiotics [2, 3]. *PDR5* has four perfect and degenerate pleiotropic drug response elements (PDREs) in its promoter region [4]. Pdr1 and Pdr3 are constitutively bound to PDRE in the *PDR5* promoter [3]. Although *PDR1* does not have a PDRE in its promoter region, *PDR3* has two PDREs, and is thereby regulated by an autoregulatory loop involving Pdr3 [5]. Thus, *PDR3* is transcriptionally regulated by both Pdr1 and Pdr3 via PDRE [5]. Gain-of-function mutations in Pdr1 and Pdr3 lead to hyperactive transcription of ABC transporter genes, such as *PDR5*, *SNQ2*, and *YOR1*, resulting in the induction of pleiotropic drug resistance (PDR) [6, 7].

Although *PDR1* and *PDR3* have functionally redundant roles in PDR, *PDR1* plays a predominant role in PDR to toxic agents and basal *PDR5* expression [8, 9]. Deletion of *PDR1* increases susceptibility to cycloheximide compared to *PDR3*, whereas the disruption of *PDR1* and *PDR3* causes hypersensitivity to cycloheximide and oligomycin compared with a single disruption [9].

*PDR3* plays a predominant role in the PDR of ρ^0/−^ cells without mitochondrial DNA in *S. cerevisiae* [10]. The retrograde signalling pathway is strongly activated in ρ^0/−^ cells of *S. cerevisiae* and *Candida glabrata*, and the expression of multidrug resistance genes, including *PDR5 and CgCdr1* is induced. The induction of *PDR5* in this retrograde signalling pathway requires Pdr3 but not Pdr1 [10]. In the retrograde signalling pathway, Pdr3 directly interacts with the Hsp70 chaperone Ssa1, and Pdr3 activity is inhibited [11]. Intriguingly, this Pdr3-Ssa1 association is decreased in ρ^0/−^ cells, and Pdr3 activity is strongly stimulated [11].

In *S. cerevisiae*, small (0.6 MDa) (Rpd3S) and large (1.2 MDa) (Rpd3L) corepressor complexes exist, sharing specific subunits such as a histone deacetylase, Rpd3, Ume1, and Sin3 [12]. They participate in chromatin remodelling and transcriptional repression [13, 14]. Dep1 and Sds3 are specific subunits of the Rpd3L complex, whereas the Rpd3S complex contains two unique subunits, Rco1p and Eaf3p [12]. Rpd3 is a histone deacetylase, while Sds3 is involved in transcriptional silencing and sporulation. The Rpd3L complex contains the DNA binding transcription factors, Ume6 and Ash1, which are responsible for the targeted deacetylation of gene promoters [12]. For example, Ume6 binds the URS1 upstream regulatory sequence on the *INO1* promoter and represses *INO1* expression by recruiting Rpd3 via the corepressor Sin3 and chromatin remodelling factor Isw2 [14, 15]. Although both Ume6 and Ash1 are bound to the *INO1* and *HO* promoters, Ume6 specifically represses *INO1* gene expression, and Ash1 specifically inhibits *HO* gene expression [12]. *UME6* is known to repress carbon/nitrogen metabolic and early meiotic gene expression while participating in gene activation [16].

Borecka-Melkusova et al. have shown that Rpd3 is required for basal *PDR5* transcription and Pdr3-mediated PDR [17]. In addition, the *sds3*Δ, *dep1*Δ, and *rpd3*Δ strains are sensitive to drugs, indicating that the Rpd3L complex is involved in PDR [17]. In contrast to *sds3*Δ, *dep1*Δ, and *rpd3*Δ strains, the *ume6*Δ and *ash1*Δ strains displayed no sensitivity to cycloheximide at the minimum inhibitory concentration [17]. In addition, Yibmantasiri et al. have also reported that the *ume6*Δ strain does not confer sensitivity to a range of fungicides including cycloheximide, ketoconazole, fluconazole, oligomycin, and benomyl when tested in a spot dilution assay [18]. The authors also reported that deletion of *UME6* does not reduce Pdr5 expression in western blot analysis [18]. Robbins et al. reported that the decreased azole resistance in the *rpd3*Δ strain of *S. cerevisiae* does not result from downregulation of *PDR5* mRNA [19]. Rather, it results from diminished Hsp90-dependent antifungal drug resistance in *Candida albicans* and *S. cerevisiae* [19]. Furthermore, Jensen et al. reported that artemisinin sensitivity in the *rpd3*Δ strain of *S. cerevisiae* occurred due to the impaired endoplasmic reticulum (ER) to Golgi trafficking of Pdr5, and not from transcriptional downregulation of *PDR5* [20].

As mentioned above, *PDR5* expression or PDR in the *rpd3*Δ, *ume6*Δ, and *ash1*Δ mutant strains, has been examined mainly in ρ^+^ cells with mitochondrial DNA, but not in ρ^0/−^ cells. However, Borecka-Melkusova et al. showed that ρ^0/−^ cells in the *rpd3*Δ strain also have significantly lower cycloheximide resistance than those in the wild-type [17]. Although *PDR5* transcription and PDR are activated by retrograde signalling via Pdr3 in ρ^0/−^ cells, whether *RPD3*, *UME6*, and *ASH1* are involved in the activation of *PDR5* transcription and PDR in ρ^0/−^ cells has not yet been examined. Therefore, this study investigated the roles of *RPD3*, *UME6*, and *ASH1* in the activation of *PDR5* transcription and PDR by retrograde signalling in ρ^0^ cells.

## Results

### Susceptibility of ρ^0^ cells in strain *ume6∆* to fluconazole and cycloheximide

**Fig. 1:**
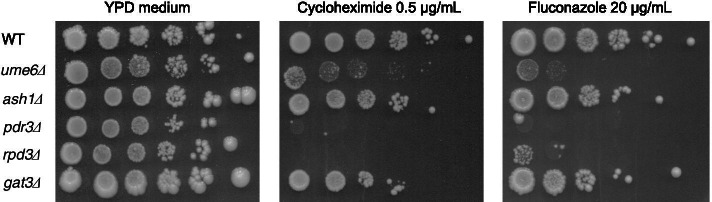
*UME6,* but not *ASH1,* is responsible for the PDR of ρ^0 ^*S. cerevisiae*. Fluconazole or cycloheximide resistance of ρ^0^ cells in the wild-type strain (FY1679-28C) and *its derivative strains, ume6*∆::*bleMX6*, *ash1*∆::*bleMX6*, *pdr3*∆::*bleMX6*, and *rpd3*∆::*bleMX6*, and *gat3*∆::*bleMX6* was determined by the spot dilution assay

To investigate whether *UME6* and *ASH1* are involved in the PDR of ρ^0^ cells, the sensitivity of ρ^0^ cells in the *ume6*∆ and *ash1*∆ mutant strains to fluconazole and cycloheximide was examined using a spot dilution assay. ρ^0^ cells in the *ume6*∆::*bleMX6*, *pdr3*∆::*bleMX6*, and *rpd3*∆::*bleMX6* mutant strains were more sensitive to fluconazole and cycloheximide than those in the wild-type, *ash1*∆::*bleMX6*, and *gat3*∆::*bleMX6* strains (Fig. 1). However, ρ^0^ cells of the *ume6*∆ mutant strain were less susceptible to fluconazole and cycloheximide than those in the *pdr3*∆ and *rpd3*∆ mutant strains (Fig. 1). Similar results were also observed for ρ^0^ cells in the wild-type, *ume6*∆::*KanMX, pdr3*∆::*KanMX*, and *rpd3*∆::*KanMX, ash1*∆::*KanMX*, and *gat3*∆::*KanMX* strains (data not shown). We also obtained similar results in ρ^0^ cells of the *ume6*∆ mutant derived from the W303–1A strain (data not shown). Since fluconazole and cycloheximide are functionally and structurally unrelated compounds, these results suggest that *UME6*, but not *ASH1*, is responsible for the activation of *PDR5* transcription and PDR in ρ^0^ cells of *S. cerevisiae*. However, it cannot be ruled out that *ASH1 may* be responsible for resistance to other drugs in ρ^0^ cells of *S. cerevisiae*.

In the spot dilution assay, the ρ^0^ cells in the *ume6*∆ mutant displayed less susceptibility to fluconazole and cycloheximide than in the *pdr3*∆ and *rpd3*∆ mutants. Therefore, we further investigated whether *UME6* is responsible for PDR in ρ^0^ cells using a co-cultivation assay [21]. As ρ^0^ cells of the *ume6*∆ mutant, but not of the *ash1*∆ and *gat3*∆ mutants, were more sensitive to fluconazole and cycloheximide than those in the wild-type strain, *ash1*∆ and *gat3*∆ mutants were used as controls for the *ume6*∆ mutant in the co-cultivation assay (Fig. 1).

**Fig. 2:**
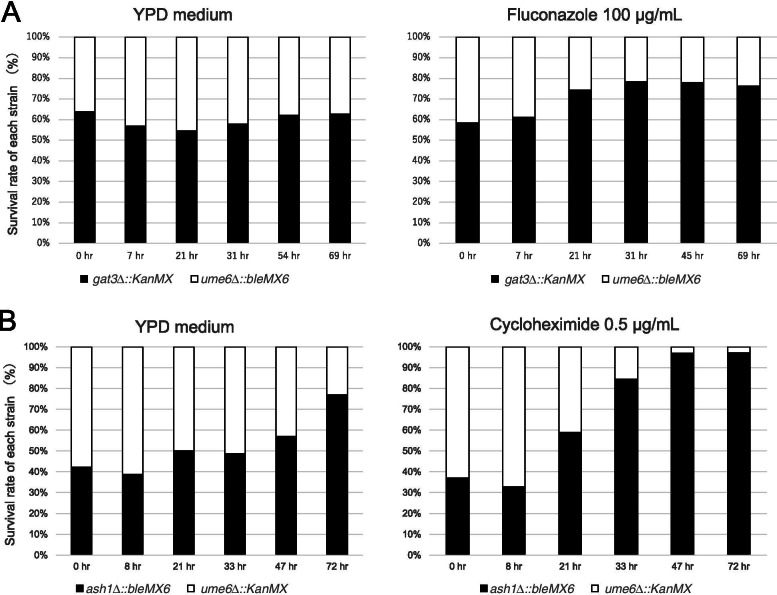
ρ^0^ cells in *ume6*∆ mutant are more susceptible to fluconazole and cycloheximide than those in the *ash1*∆ and *gat3*∆ mutants in the co-cultivation assay. **A** Changes in the survival rate of each strain in the co-cultivation of *ume6*∆::*bleMX6 and gat3*∆::*KanMX* strains (FY1679-28C) in the presence (right) and absence (left) of 100 μg/mL fluconazole. **B** Changes in the survival rate of each strain in the co-cultivation of *ash1*∆::*bleMX6 and ume6*∆::*KanMX* strains (FY1679-28C) in the presence (right) and absence (left) of cycloheximide (0.5 μg/mL)

ρ^0^ cells in two mutant strains, *gat3*∆::*KanMX* and *ume6*∆::*bleMX6,* were co-cultivated in the presence and absence of 100 μg/mL fluconazole. The number of viable cells of each mutant strain in the co-culture was estimated by spreading them onto yeast extract peptone dextrose (YPD) plates containing G418 or Zeocin. We found that ρ^0^ cells in the *ume6*∆::*bleMX6* strain were eliminated from the co-culture over time in the presence of fluconazole, but not in the absence of fluconazole (*p* < 0.05) (Fig. 2A). Similar results were also observed when ρ^0^ cells of the *gat3*∆::*bleMX6* strain were co-cultivated with those of *ume6*∆::*KanMX* strain in the presence and absence of 100 μg/mL fluconazole, indicating that selection marker genes do not affect these changes in survival rate (data not shown). Furthermore, similar results were obtained when ρ^0^ cells of the *gat3*∆ strain were co-cultivated with those of *ume6*∆ strain in the presence and absence of 0.5 μg/mL cycloheximide (data not shown). Rather than using the *gat3*∆ strain, ρ^0^ cells of the *ash1*∆::*bleMX6* strain were co-cultivated with those of *ume6*∆::*KanMX* in the presence and absence of 0.5 μg/mL cycloheximide. The survival rate of each strain in the co-culture was estimated in the same way. Consequently, ρ^0^ cells in the *ume6*∆::*KanMX* strain were eliminated earlier from the co-culture over time in the presence of cycloheximide compared to that in the absence of cycloheximide (*p* < 0.05) (Fig. 2B). Furthermore, similar results were observed in the co-cultivation of ρ^0^ cells in the *ash1*∆::*KanMX* and *ume6*∆::*bleMX6* mutant strains in the presence of 100 μg/mL fluconazole (data not shown). These results suggest that *UME6*, but not *ASH1*, contributes to the activated PDR in ρ^0 ^ cells of *S. cerevisiae*.

### Deletion of *RPD3* and *UME6*, but not *ASH1*, decreases the *PDR5* mRNA level

Decreased PDR in ρ^0^ cells of the *rpd3*∆ and *ume6*∆ strains suggested that enhanced transcriptional expression of *PDR5* by retrograde signalling is suppressed in ρ^0^ cells. Thus, we investigated the expression levels of *PDR5* mRNA in ρ^0^ and ρ^+^ cells of the wild-type, *rpd3*∆, *ume6*∆, *pdr3*∆, *ash1*∆ mutant strains grown to the logarithmic phase by real-time RT-PCR.

**Fig. 3:**
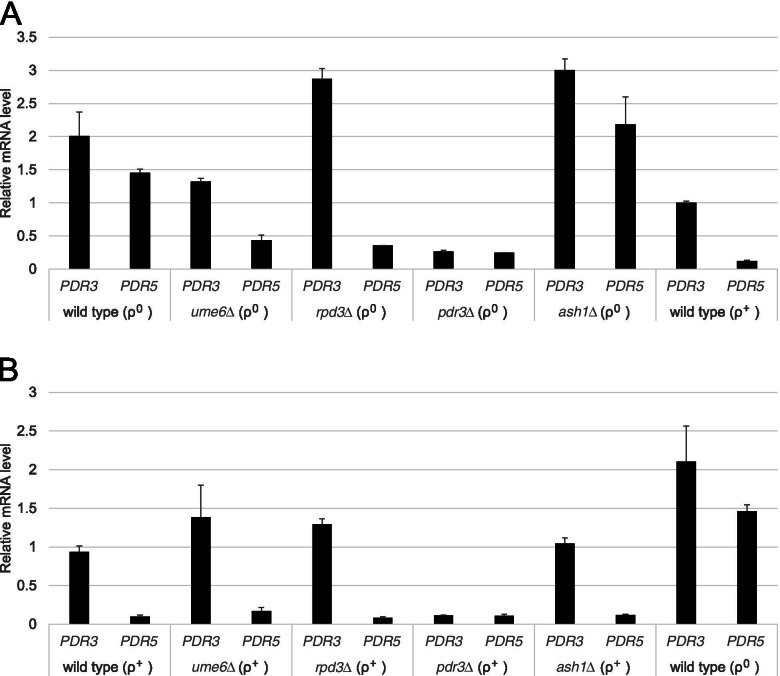
Transcriptional expression levels in the logarithmic growth phase of *PDR3* and *PDR5* in ρ^0^ and ρ^+^ cells of the wild-type, *rpd3*∆, *ume6*∆, *pdr3*∆, *ash1*∆ mutant strains. **A** Relative *PDR3* and *PDR5* mRNA levels in ρ^0^ cells of the wild-type, *ume6*∆, *rpd3*∆, *pdr3*∆, *ash1*∆ mutant strains in the logarithmic growth phase were determined by qRT-PCR. **B** Relative *PDR3* and *PDR5* mRNA levels in ρ^+^ cells of the wild-type, *ume6*∆, *rpd3*∆, *pdr3*∆, *ash1*∆ mutant strains in the logarithmic growth phase were determined by qRT-PCR

*PDR5* expression was more strongly induced in the wild-type strain of ρ^0^ cells than in the wild-type strain of ρ^+^ cells (*p* < 0.05) (Fig. 3A and Table S1). Within ρ^0^ cells, *PDR5* expression of the *pdr3*∆ strain was significantly more suppressed compared to the wild-type strain (*p* < 0.05), while in case of ρ^+^ cells, the *PDR5* expression level in the *pdr3*∆ strain was not significantly different from the wild-type strain (*p* > 0.05) (Fig. 3B and Table S2). These results were consistent with those of previous reports [9].

**Fig. 4:**
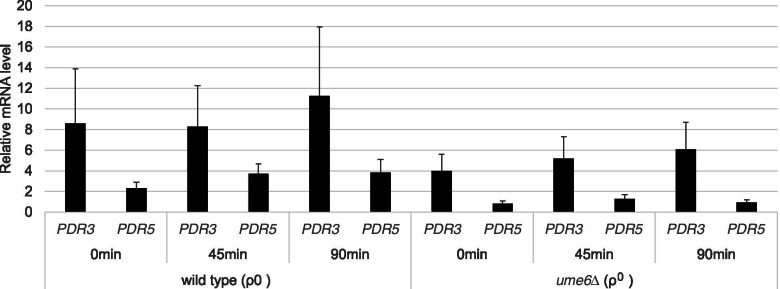
Transcriptional expression changes *of PDR3* and *PDR5* in ρ^0^ cells of the wild-type and *ume6*∆ strains when cycloheximide was added. *PDR3* and *PDR5* mRNA levels in ρ^0^ cells of the wild-type and *ume6*∆ strains at 45 min and 90 min after addition of cycloheximide (0.2 μg/mL) were quantified by qRT-PCR

The *PDR5* mRNA levels in ρ^0^ cells of the *rpd3*∆, *ume6*∆, and *pdr3*∆ mutant strains were significantly lower than those in the wild-type and *ash1*∆ mutant strains (*p* < 0.05) (Fig. 3A and Table S1). This suggests that activated *PDR5* transcriptional expression by retrograde signalling was significantly reduced in ρ^0^ cells of the *rpd3*∆, *ume6*∆, and *pdr3*∆ strains (Fig. 3A). Thus, the increased drug susceptibility of ρ^0^ cells in the *rpd3*∆, *ume6*∆, and *pdr3*∆ strains shown in spot dilution and co-cultivation assays can be explained by the reduction in *PDR5* mRNA levels. In contrast, *PDR5* mRNA levels in ρ^+^ cells of the *ume6*∆, *rpd3*∆, and *pdr3*∆ strains were similar to those in ρ^+^ cells of the wild-type and *ash1*∆ mutant strains (*p* > 0.05) (Fig. 3B and Table S2). Therefore, *UME6* and *RPD3* are responsible for the enhanced transcriptional expression of *PDR5* by Pdr3-mediated retrograde signalling in ρ^0^ cells but not for basal expression of *PDR5* in ρ^+^ cells. In addition, ρ^0^ cells, but not ρ^+^ cells, in the *ume6*∆ mutant strain, had slightly more reduced *PDR3* mRNA levels than those in the wild-type strain, suggesting minor involvement of *UME6* in the activation of autoregulated transcriptional expression of *PDR3* by retrograde signalling (Figs. 3A and 4).

Furthermore, this study investigated whether the activated transcriptional expression of *PDR5* following cycloheximide exposure occurs in ρ^0^ cells of the u*me6*∆ mutant strain using real-time RT-PCR. *PDR3 and PDR5* mRNA levels in ρ^0^ cells of the *ume6*∆ strain were lower than those in the wild-type strain, independent of the addition of cycloheximide. However, *PDR5* mRNA levels in ρ^0^ cells of the wild-type and *ume6*∆ strains increased 1.6- and 1.55 times, respectively, after exposure to 0.2 μg/mL cycloheximide for 45 min (Fig. 4 and Table S3). These increases in *PDR5* mRNA levels in ρ^0^ cells were statistically significant in the wild-type (*p* < 0.05), but not in the *ume6*∆ strains (*p* > 0.05). This suggests that *UME6* is involved in the transcriptional expression of *PDR5* in cycloheximide-exposed and unexposed ρ^0^ cells and the intact induction of *PDR5* transcription after drug exposure in ρ^0^ cells.

## Discussion

This study revealed that *UME6* and *RPD3,* but not *ASH1,* are responsible for enhancing *PDR5* expression and PDR by retrograde signalling in ρ^0^ cells of *S. cerevisiae*. In addition, *UME6* was involved in the transcriptional expression of *PDR5* in cycloheximide-exposed and unexposed ρ^0^ cells, and the enhancement of *PDR5* transcription after cycloheximide exposure was also impaired in ρ^0^ cells of the *ume6*∆ mutant strain. Reduced *PDR5* mRNA levels in the presence and absence of cycloheximide were also reported in ρ^+^ cells of the *rpd3*∆ strain by Borecka-Melkusova et al.; however, this report was invalidated later by Robbins et al. [17, 19]. Histone deacetylation leads to transcriptional repression and activation [14, 22]. Thus, Ume6 and Rpd3 may serve as enhancers of *PDR5* expression by retrograde signalling in ρ^0^ cells, different from their usual roles as repressors.

It is currently unknown how Rpd3 and Ume6 enhance the transcriptional expression of *PDR5* and PDR in ρ^0^ cells and why they do not affect basal *PDR5* expression in ρ^+^ cells. As Ume6 binds to the *PDR5* promoter region in ρ^+^ cells, it may also be localised at the *PDR5* promoter region in ρ^0^ cells [23]. If this is true, Rpd3 and Ume6 may directly mediate the activation of *PDR5* expression by chromatin remodelling and facilitating Pdr3 binding. Furthermore, the enhanced *PDR5* transcription and PDR by Rpd3 and Ume6 in ρ^0^ cells may be indirectly caused by changes in the expression of other genes. Pdr1 and Pdr3 can bind to the KIX domain of the transcriptional Mediator subunit Med15/Gal11, which mediates sequence-specific transcriptional regulatory proteins and the RNA polymerase II machinery [24]. L-Mediator (of the Mediator complexes) contains the Cdk8 subcomplex, which consists of the cyclin-dependent kinase Cdk8 (Med15/Srb8), Med12 (Srb8), and Med13 (Srb9). Deletion of Med12 from the Cdk8 complex completely suppressed the induction of *PDR5* expression in ρ^0^ cells but not in ρ^+^ cells, indicating a difference in the regulatory machinery of *PDR5* transcription between ρ^+^ and ρ^0^ cells [25]. This difference may be associated with the difference in transcriptional regulation of *PDR5* by Rpd3 and Ume6 between ρ^0^ and ρ^+^ cells.

This study showed that ρ^0^ cells in the *ume6*∆ mutant were less susceptible to fluconazole and cycloheximide than in the *pdr3*∆ and *rpd3*∆ mutants as assessed by the spot dilution assay (Fig. 1). This suggests that ρ^0^ cells in the *ume6*∆ mutant, but not the *rpd3*∆ mutant, maintain Hsp90-dependent antifungal drug resistance and intact ER to Golgi trafficking of Pdr5 [19, 20]. Furthermore, fewer multidrug resistance genes other than *PDR5* are downregulated in ρ^0^ cells of the *ume6*∆ mutant than in those of the *pdr3*∆ mutant [26].

ρ^0^ cells, but not ρ^+^ cells, in the *ume6*∆ mutant strain had slightly lower *PDR3* mRNA levels than those in the wild-type strain (Figs. 3 and 4). Ume6 binds to the *PDR3* promoter region in ρ^+^ cells; therefore, it may also bind to the *PDR3* promoter region in ρ^0^ cells and may directly activate the transcriptionally auto regulated loop of *PDR3* by chromatin remodelling and facilitating Pdr3 binding [23].

The emergence of multidrug-resistant fungi is a serious clinical concern [27]. Therefore, the efficacy of combined antifungal agents against multidrug-resistant fungi has been examined [28]. Furthermore, to treat multidrug-resistant fungal infections, the efficacy of using histone deacetylase inhibitors or essential oils from plants in combination with the primary classes of antifungals has also been examined [29–35]. For example, a histone deacetylase inhibitor, trichostatin A, decreases the upregulation of *CDR1*, *ERG1*, and *ERG11* by azole and enhances azole sensitivity in *C. albicans* [33]. Uracil-based histone deacetylase inhibitors 1c and 1d reduce acquired resistance to antifungals and trailing growth in *C. albicans* [34]. In addition, *RPD3* is responsible for azole resistance and basal transcription of efflux genes such as *CDR1*, *CDR2*, and *MDR1* in ρ^+^ cells of pathogenic *C. albicans* [35]. Thus, Ume6 may also be responsible for multidrug resistance via transcriptional regulation of the efflux genes in ρ^+^ and ρ^0^ cells of pathogenic *Candida* species. Therefore, identifying specific inhibitors of Ume6 may lead to the development of drugs against multidrug-resistant pathogenic *Candida* species.

## Conclusions

*PDR5* expression or PDR in the *rpd3*Δ, *ume6*Δ, or *ash1*Δ mutant strains has been examined primarily in ρ^+^ cells, but not in ρ^0/−^ cells. In this study, we investigated the roles of *RPD3*, *UME6*, and *ASH1* in the activation of *PDR5* transcription and PDR by retrograde signalling in ρ^0^ cells. Using spot dilution and co-cultivation assays, we have shown that *RPD3* and *UME6*, but not *ASH1*, contribute to the PDR in ρ^0^ cells of *S. cerevisiae*. In addition, using real-time PCR assay, we have shown that *RPD3* and *UME6*, but not *ASH1*, are involved in the transcriptional expression of *PDR5* in ρ^0^ cells, and *UME6* also contributes to *PDR5* transcription and its enhancement in cycloheximide-exposed ρ^0^ cells. This work provides useful knowledge on the genetic basis of yeast multidrug resistance via transcriptional regulation of efflux genes.

## Methods

### Yeast strains and media

FY1679-28C (MATa, ura3–52, leu2-D1, trp1-D63, his3-D200, GAL2+) and W303–1A (MATa, ura3–1, leu2–3,112, trp1–1, his3–11,15, ade2–1, can1–100, rad5–535) strains were used as wild-type strains [27]. W303–1A was provided by the National Bio-Resource Project, Japan. To construct the gene deletion mutant strains, open reading frames of *UME6*, *PDR3*, *RPD3*, *ASH1*, or *GAT3* were replaced with *KanMX* or *bleMX6* gene cassettes by PCR-mediated one-step gene disruption in the FY1679-28C or W303–1A background [36].

The strains described above were grown on glycerol-rich YPG agar plates (2% glycerol, 1% yeast extract, 2% bactopeptone, 2% agar) to eliminate ρ^0^ cells and obtain ρ^+^ cells for real-time RT-PCR [17]. The ρ^0^ derivatives of the strains described above were obtained by plating the cells twice on YPD agar plates (2% glucose, 1% yeast extract, 2% bactopeptone, and 2% agar) containing 40 μg/ml ethidium bromide [26].

Yeast cells were grown in YPD medium (2% glucose, 1% yeast extract, and 2% bactopeptone) at 30 °C with shaking.

### Spot dilution assay

A spot dilution assay was conducted to estimate the relative resistance of each yeast strain to fluconazole or cycloheximide [37, 38]. Three independently derived ρ^0^ cells from each yeast strain were aerobically grown to an OD_600_ of 0.6–0.9 at 30 °C in YPD medium. Five microliters of 10-fold serial dilutions of the logarithmic phase cultures containing the same number of cells were spotted on YPD plates containing or not containing 20 μg/mL fluconazole (Nacalai Tesque) (or 0.5 μg/mL cycloheximide (Wako)) and incubated at 30 °C for 7 days. Representative plate images of three replicates were captured after culturing at 30 °C for 7 days.

### Co-cultivation of two gene deletion mutants replaced with *KanMX* or *bleMX6* gene cassettes

The *ume6*∆::*KanMX* and *ash1*∆::*bleMX6* mutant strains, or the *ume6*∆::*bleMX6* and *ash1*∆::*KanMX* mutant strains were co-cultivated in YPD medium containing or not containing 100 μg/mL fluconazole (or 0.5 μg/mL cycloheximide). The aliquots of the co-culture were recovered immediately before adding drugs and at various times after addition of the drugs, and spread on the YPD plates containing G418 (Wako) or Zeocin (Nacalai Tesque) [20]. The viability of each strain at each time point was estimated from the colony numbers on G418 and Zeocin plates [20]. The previous experiments were also performed in the *ume6*∆::*KanMX* and *gat3*∆::*bleMX6* mutant strains, or the *ume6*∆::*bleMX6* and *gat3*∆::*KanMX* mutant strains.

### RNA extraction from ρ^0^ and ρ^+^ cells of each mutant strain grown to the logarithmic phase

Two independently derived ρ^0^ and ρ^+^ cells from each yeast strain were grown to an OD_600_ of 7–9 in YPD, diluted to an OD_600_ of 0.2, and grown for an additional 5 h in duplicate [39, 40]. Aliquots of the duplicates were recovered. The cells in the aliquots above were pelleted, washed, frozen at − 80 °C, and used to extract total RNA [39, 40]. Total RNA was isolated from yeast cells using Nucleospin RNA Plus (TaKaRa), according to the manufacturer’s protocol.

### RNA extraction from ρ^0^ cells exposed to drug

Independently derived ρ^0^ cells from each yeast strain were grown to an OD_600_ of 7–9 in YPD, diluted to an OD_600_ of 0.2, and grown for an additional 5 h in triplicate [39, 40]. Aliquots of the triplicates were harvested just before the addition of cycloheximide to the medium. Cycloheximide (0.2 μg/mL) was added to one of the triplicates, and the triplicates were grown for 45 min and 90 min at 30 °C. Aliquots of the triplicates were recovered at 45 min and 90 min after adding cycloheximide to one of the triplicates. The cells in the aliquots above were pelleted, washed, frozen at − 80 °C, and used to extract total RNA [39, 40]. Total RNA was isolated from yeast cells before and after exposure to cycloheximide using Nucleospin RNA Plus (TaKaRa), according to the manufacturer’s protocol.

### Real-time RT-PCR

Reverse transcription of total RNA was performed using the PrimeScript 1st strand cDNA Synthesis Kit (TaKaRa). SYBR Green qRT-PCR was performed using the TB Green® Premix Ex Taq II (TaKaRa) in a Step One Real-time PCR system (Applied Biosystems). For quantitative PCR analysis, the housekeeping gene *ACT1* was used as an endogenous control to normalise the expression level of each target gene [41]. Minus reverse transcriptase control was used as the negative control. qPCR for each sample was performed in duplicate or triplicate. Serial dilutions of the control cDNA from the wild-type strain were prepared to produce a standard curve for each primer pair. The primers used for *PDR3* were: forward, 5′-TACCGCAGAAGGAGGATAGTTCCCA-3′ and reverse, 5′-GCTTAATCGCAGTGTCCAGATGCTGTAC-3′, yielding a PCR product of 117 bp. The qPCR for *PDR5* was performed using primers 5′-CTCTGAGAGAACCCTGAACAAAGATATGCTA-3′ (forward) and 5′-ATAGCTTCACGGCTTGCTTCATCGT-3′ (reverse) to amplify a fragment of 165 bp. The primers used for *ACT1* were as follows: forward, 5′-CAAATTATGTTTGAAACTTTCAACGTTCCAG-3′ and reverse, 5′-ACGTGAGTAACACCATCACCGGAATC-3′, yielding a PCR product of 125 bp.

### Statistical analysis

The survival rate of *ume6*∆ strain at each time point in Fig. 2 was normalized by that at 0 h. Paired *t*-test was used for statistical analysis in Figs. 2 and 4. Unpaired Student’s *t*-test was used for statistical analysis in Fig. 3. *p* < 0.05 was considered significant.

## Supplementary Information

**Additional file 1: Table S1.** Relative expression levels quantified by qRT-PCR in Fig. 3A.

**Additional file 2: Table S2.** Relative expression levels quantified by qRT-PCR in Fig. 3B.

**Additional file 3: Table S3.** Relative expression levels quantified by qRT-PCR in Fig. 4.

## Data Availability

The datasets used and/or analyzed during the current study are within the manuscript and the Additional files.

## Abbreviations

ABC: ATP-binding cassette
PDR: Pleiotropic drug resistance
ER: Endoplasmic reticulum
PDRE: Pleiotropic drug response elements
